# Peer Review of “Influence of the COVID-19 Lockdown on the Physical and Psychosocial Well-being and Work Productivity of Remote Workers: Cross-sectional Correlational Study”

**DOI:** 10.2196/34608

**Published:** 2021-12-01

**Authors:** Laura Taraboanta

**Affiliations:** 1 Click Therapeutics New York, NY United States

*This is a peer-review report submitted for the paper “Influence of the COVID-19 Lockdown on the Physical and Psychosocial Well-being and Work Productivity of Remote Workers: Cross-sectional Correlational Study”.*

## Round 1 Review:

### General Comments

No major comments to add. The overall design, analysis, and conclusion [1] present a critical view into the impact of the COVID-19 pandemic on remote workers in the UK and help facilitate broader conversations on how to continue to track physical and psychological metrics as restrictions are easing. Additionally, the paper offers recommendations on how employers and government policy and guidelines can best support overall well-being and productivity by looking at the data and seeing where the unmet needs are. A follow-up study would be interesting with the same participants, tracking them over the span of 1 year to 18 months and assessing the same metrics to look for any change in the scores (improvements, further decline, etc).
